# Radiation incident learning in multi‐site centers

**DOI:** 10.1002/acm2.70369

**Published:** 2025-11-18

**Authors:** Xing Li, Fan‐Chi Su, Qiongge Li, Jiajin Fan, Ashish Chawla, Ann Miner, Robabeh Rahimi

**Affiliations:** ^1^ Department of Radiation Oncology Loyola University Chicago Stritch School of Medicine Maywood Illinois USA; ^2^ Department of Advanced Radiation and Proton Therapy Inova Schar Cancer Institute Fairfax Virginia USA; ^3^ Department of Radiation Oncology University of Texas Southwestern Medical Center Dallas Texas USA; ^4^ Department of Radiation Oncology University of Maryland School of Medicine Baltimore Maryland USA

**Keywords:** Muti‐site clinics, Proactive risk management, Radiation incident learning

## Abstract

**Purpose:**

Incident learning in large, multi‐site hospital systems faces challenges from resource constraints, diverse site cultures, and deviations from system‐wide policies. This study introduces a novel, resource‐efficient hybrid approach to facilitate cross‐site learning and proactive risk management, ultimately enhancing patient safety.

**Methods:**

A hybrid approach, integrating statistical, and root cause analyses was used to analyze safety events recorded from 2023 to 2024 at five sites. Events were categorized by time, site, type, and harm score with statistics tracking the event frequency per category. Failure modes (FMs) were identified with summative keywords. The frequency and cross‐site correlation of keywords were innovatively displayed by word clouds. The hybrid approach was exemplified at a high‐throughput satellite clinic, where treatments and incidents were tracked quarterly. A proactive model was also employed to predict the number of potentially affected patients at this site.

**Results:**

An analysis of 228 reported events revealed that most incidents occurred within the treatment planning category, where the keywords “carepath” and “contour” were identified as the top two frequently reported FMs, repeated 13 and 10 times respectively. The keywords “contour” and “documentation” appeared across four sites, followed by “carepath” and “couch” which appeared across three sites. At the featured satellite clinic, a total of 18 FMs were identified from yearly 8,714 treatments. The proactive model showed an average risk priority number decreased from 106 to 77, and the estimated number of affected patients decreased from 27 to 20 at this site.

**Conclusions:**

We developed a comprehensive, system‐wide approach that enhanced efficiency in incident learning across multi‐site clinics. Site‐specific shifts in safety culture were effectively benchmarked and cross‐site learning was facilitated through measurable endpoints. A novel word cloud analysis was developed, enhancing visualization of cross‐site root causes. Additionally, a proactive model was adopted to increase system‐wide preparedness and improve patient safety outcomes.

## 1 INTRODUCTION

Incident learning in modern radiation therapy is complex, driven by advanced techniques and intricate workflows that demand high expertise and coordinated teamwork. As cancer treatment evolves toward integrated approaches, involving patient transfers across modalities and sites, the demand for staff cross‐coverage and synchronized management intensifies. Such evolution necessitates streamlined processes, consistent system‐wide policies, and clear communication to ensure patient safety.

An impactful incident learning mechanism begins by cultivating a positive safety culture,^1^ which enables staff to easily report incidents and receive timely feedback. A structured Incident Learning System (ILS)^2^, ^3^, ^4^ that centrally records incidents is a crucial tool to support the culture. Systems such as the Radiation Oncology Incident Learning System (RO‐ILS)^5^, ^6^ are prevalent and internal ILSs have also been widely adopted by many health systems to enhance safety and comply with regulatory mandates.

Subsequently, establishing an effective mechanism for incident analysis is critical. Tracking incident occurrences is a common method to benchmark shifts in safety culture^7^ or assess the effectiveness of specific tools.^8^, ^9^ However, this statistical approach, while intuitive may harbor inherent limitations, as occurrences do not always accurately reflect outcomes.^10^, ^11^ The limitations can be pronounced in multi‐site clinical settings due to variations in staffing levels, staff experience, and patient loads across sites, leading to inaccurate analyses when reliance is solely placed on occurrences.

Failure Modes and Effects Analysis (FMEA) utilizes three parameters—Severity (S), Occurrence (O), and Detectability (D)^12^ to address the limitations of an occurrence‐only approach. These parameters are multiplied to obtain the Risk Priority Number (RPN) to prioritize failure modes (FMs) and quantify the overall impact. FMEA has proven effective in identifying failure points and establishing safety barriers for localized challenges, such as those in treatment planning^13^, ^14^ spanning across external beam radiation therapy (EBRT),^15^ brachytherapy,^16^ and proton therapy.^17^ FMEA requires a detailed breakdown of clinical workflows into multi‐layered flowcharts. Each step is assessed with an RPN. The process involves a multidisciplinary team, including radiation oncologists, medical physicists, dosimetrists, and therapists. Depending on the clinical setting, the team may also include nurses, billing staff, front desk personnel, and IT specialists. All participants contribute their expertise,  and collectively the team thoroughly understands the entire process to ensure unbiased risk assessments. Consequently, the substantial time and resource demands pose significant challenges for FMEA‐based incident learning within large hospital systems.

The challenges of implementing FMEA in multi‐site clinics are magnified by factors such as large data cohorts, diverse site cultures, different machine configurations and modalities, and deviations from system‐wide policies. Therefore, more resources are required to uphold consistent standards and promote sustainable improvements across the network, underscoring the importance of efficiency. Recently, researchers have developed a more efficient hybrid method that integrates FMEA with data‐driven techniques to enhance safety and quality in treatment planning.^18^ This approach has demonstrated its potential to be generalized for incident learning across multi‐site clinics. To further improve efficiency, it is essential to promote cross‐site learning with measurable endpoints. Recent advancements in FMEA research have led to a proactive model that leverages existing site‐specific data to predict such endpoints,^19^, ^20^ offering quantifiable improvements that could also transform the safety culture.

In this study, we present our institution‐specific experience in conducting radiation incident learning across multiple clinical sites. We developed a hybrid approach that integrated statistical and FMEA‐based models, demonstrating its effectiveness and efficiency in facilitating incident learning and proactive risk management within multi‐site clinics.

## 2 MATERIALS AND METHODS

### 2.1 Multi‐site settings

Our health system operates a network of five clinics, including a main center and four satellite centers, collectively treating from 250 to 300 patients daily. In 2023 and 2024, approximately 3,800 to 4,100 new patients were treated each year. Our facilities offer a comprehensive range of external beam radiation therapy (EBRT) treatment modalities across the network, including specialized procedures such as stereotactic radiosurgery (SRS), stereotactic body radiotherapy (SBRT), total skin electron therapy (TSET), and total body irradiation (TBI).

The main center is equipped with two TrueBeam linear accelerators (linacs), a CyberKnife linac and an Ethos 2.0 linac currently being installed. Additionally, it houses a two‐room IBA Proteus Plus proton therapy unit and maintains an active brachytherapy program, offering high‐dose rate (HDR) and low‐dose rate (LDR) brachytherapy treatments. The satellite clinics house six linacs, including four TrueBeam linacs, two Trilogy linacs, and a Halcyon 4.0 linac equipped with HyperSight image‐guided radiotherapy (IGRT) capabilities. HDR and LDR brachytherapy treatments are available at two of the satellite sites. All centers utilize ARIA record and verify (R&V) system, along with Eclipse and RayStation treatment planning systems (TPS) for EBRT planning, and Oncentra TPS for brachytherapy planning. Table 1 provides a summary of the clinical services and equipment across all sites.

**TABLE 1:** Clinical services, equipment, and patient load across sites.

Site	Services	Equipment	^*^Patient load
A	EBRT, SBRT, SRS, TBI, TSET, HDR‐BT, LDR‐BT, proton therapy	TrueBeam, CyberKnife, Ethos 2.0, IBA ProteusPLUS, Flexitron HDR afterloader	125 ‐ 150
B	EBRT, SBRT, HDR‐BT	TrueBeam, Halcyon 4.0, Flexitron HDR afterloader	40 – 50
C	EBRT, SBRT, HDR‐BT	TrueBeam, Trilogy, Flexitron HDR afterloader	30 – 35
D	EBRT, SBRT	TrueBeam, Trilogy	35 – 40
E	EBRT, SBRT	TrueBeam	20 – 25

* Patient load was estimated from the average daily patient numbers per site during the study period.

Abbreviations: EBRT = External Beam Radiation Therapy; SBRT = Stereotactic Body Radiation Therapy; SRS = Stereotactic Radiosurgery; TBI = Total Body Irradiation; TSET = Total Skin Electron Therapy; HDR‐BT = High‐dose‐rate brachytherapy; LDR = Low‐dose‐rate brachytherapy

### 2.2 Incident reporting and review

Safety incidents were centrally collected through a hospital‐wide online platform accessible to all employees, who must complete mandatory trainings on its use during new employee orientation. Following recommendations from existing literature,^21^ we established a radiation safety incident learning committee to conduct bi‐weekly incident reviews. The core committee members included the Department Chair (radiation oncologist), the Director of the Cancer Center, a Chief Physicist, a Lead Physicist for proton therapy, a Lead Physicist for brachytherapy, a Chief Dosimetrist, a Chief Therapist, Managers, Lead physicists and Lead therapists from four satellite clinics, and a billing team leader. All reported events were reviewed and the committee determined when ad hoc breakout meetings were required for further in‐depth reviews. For each incident, a committee member was appointed as the case owner, responsible for follow‐up with the reporter and providing a feedback loop to the committee.

Incidents were classified into four categories: event time, event site, event type, and harm score. The harm score, a mandatory category within the internal reporting system, included four levels: No Harm, Mild/Temporary Harm, Severe/Permanent Harm, and Death. Reporters assigned an initial harm score, with the final score determined through committee review and consensus. We identified five types of events: simulation, treatment planning, treatment delivery, operation, and others, as detailed in Table 2. The categorized event types were cross‐checked by three board‐certified physicists with varied clinical expertise and experience: a proton physicist with seven years, a photon physicist with nine years, and a senior physicist with fifteen years of experience.

**TABLE 2:** Classification of Event Types.

Event Type	Description
Simulation	Events occurred during CT or MRI simulations, excluding IGRT, which is categorized under Treatment Delivery.
Treatment Planning	Events occurred during the treatment planning phase, including prescription and contour errors.
Treatment Delivery	Events specific to the delivery of treatment.
Operation	Events not related to simulation, treatment planning and treatment delivery, such as patient scheduling, patient consent, equipment malfunctions, sterilization errors, and quality assurance issues.
Others	Events erroneously reported to the radiation oncology department from other departments, or any non‐treatment‐related patient emergencies, such as falls.

### 2.3 Risk assessment

The hybrid approach combined statistical and root cause analyses to assess the safety events recorded between 2023 and 2024, which were systematically categorized based on the criteria introduced in Section 2.2 and identified with failure modes (FMs). For each FM, a keyword summarizing the root cause was assigned. To ensure consistency, keyword assignment was restricted to the three physicists who categorized the events, with final keywords determined through consensus among them. Subsequently, the frequency of keyword was calculated as the ratio of the event counts per keyword to the total event counts. To facilitate visualization across clinical sites, word clouds were generated using Equation (1), where the font size of a keyword is proportional to its frequency with larger sizes indicating more frequent occurrences:

(1) $$\begin{array}{c} F\left(w_{i}\right)=wF\left(w_{\max}\right)+\left(1-w\right)F\left(w_{\min}\right)\\ w=\frac{f\left(w_{i}\right)-f\left(w_{\min}\right)}{f\left(w_{\max}\right)-f\left(w_{\min}\right)} \end{array}$$

where 𝓌 is the weighing factor. $F(w_{i})$ and $f(w_{i})$ specify the font size and the appearance frequency of keyword $w_{i}$, respectively, with $F(w_{\min})$ and $F(w_{\max})$ representing the minimum and maximum font sizes, respectively, and $f(w_{\min})$ and $f(w_{\max})$ representing the minimum and maximum appearance frequencies, respectively. The overlaps of keywords across multiple sites were assessed and the repeated keywords were labelled with the same color.

Site B was selected for proactive risk assessment due to its high patient treatment volume per machine and its diverse range of modalities, which include two linacs, an HDR unit, and a CT simulator, representing a typical satellite clinical setting. Traditional FMEA was performed by evaluating the S‐, O‐, and D‐values, and calculating the corresponding RPN values, following the American Association of Physicists in Medicine (AAPM) Task Group 100 (TG‐100) guideline.^12^ A proactive model, which was adapted from recent research advancements,^19^, ^20^ was also applied to estimate the number of potentially affected patients using Equation (2), where *N* represents the impacted patients, $N_{\mathrm{total}}$ is the total patient throughput, and P(O) and P(D) are the probabilities of O and D, respectively determined from Table 2 in the TG‐100 report.

(2) $$N=N_{\mathrm{total}}\times P\left(O\right)\times P\left(D\right)$$

## 3 RESULTS

### 3.1 Multi‐site incident learning

**FIGURE 1:**
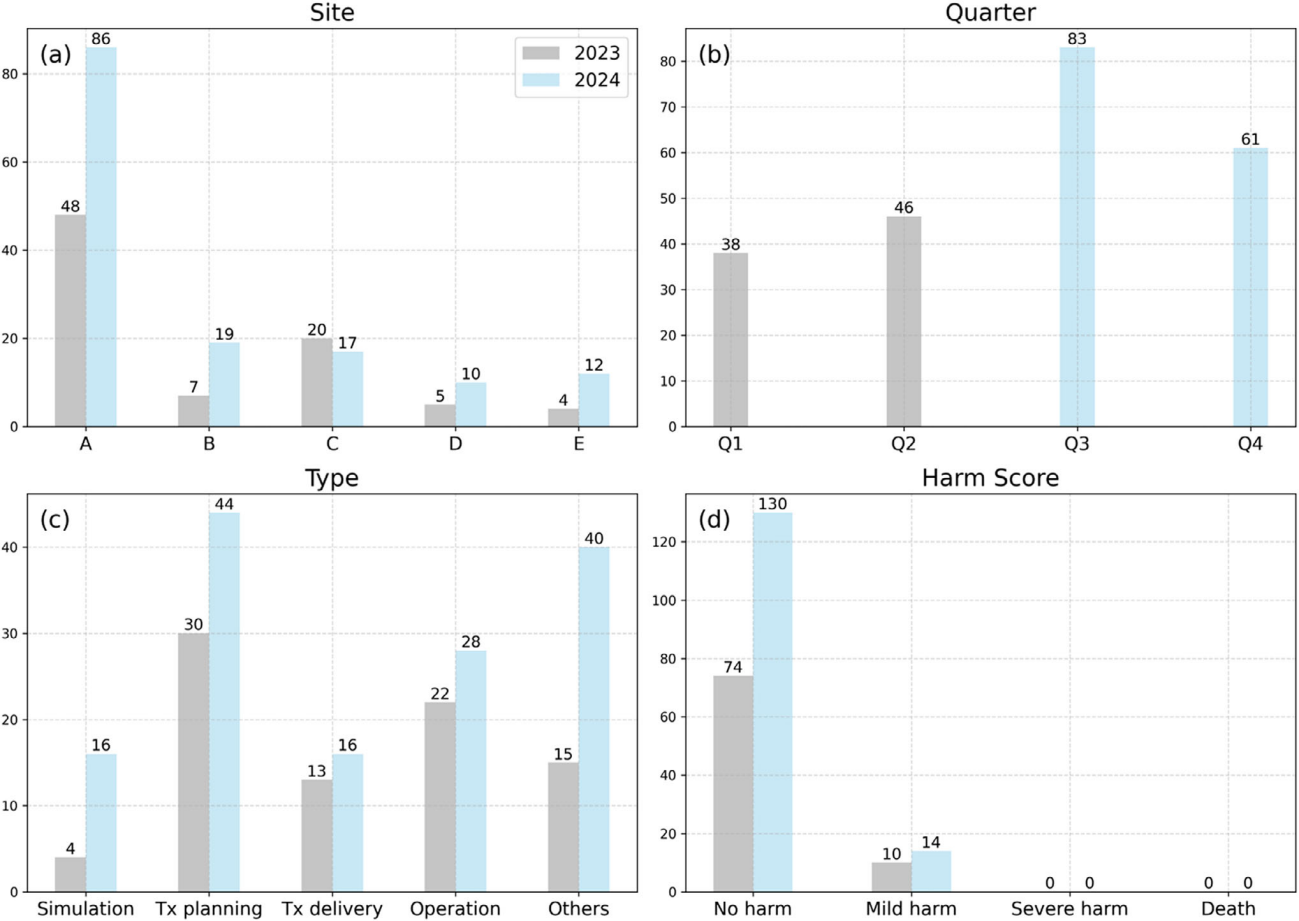
Multi‐site statistics. Data were recorded from July to December 2023 (in gray) and from January to June 2024 (in cyan). (a) Incident counts per clinical site, with site A representing the main center and sites B to E representing satellite centers. (b) Incident counts per quarter (Q), aligned with the study period: Q1 (July to September 2023), Q2 (October to December 2023), Q3 (January to March 2024), and Q4 (April to June 2024). (c) Incident counts categorized by process type as defined in Table 1. (d) Incident counts classified by harm score.

Figure 1 presents a statistical analysis of 228 reported incidents from July 2023 to June 2024, categorized by site, type, time, and harm score. Site A, the main center, exhibited the most notable increase in incident counts, from 48 in 2023 to 86 in 2024 (+79%). Sites B, D, and E also reported increases, with Site B rising from 7 to 19 (+171%), Site D from 5 to 10 (+100%), and Site E from 4 to 12 (+200%). In contrast, Site C reported a slight reduction in incidents from 20 to 17 (−15%). The first quarter of the study Q1 (July to September 2023) recorded the fewest incidents, 38 (16%), whereas the third quarter Q3 (January to March 2024) recorded the most, 83 (36%). Regarding event types, the highest number of incidents occurred in “Treatment Planning” (32%), followed by “Others” (24%), and “Operation” (22%). Most incidents were categorized as “No harm” (89%), with 11% as “Mild harm”, and none resulting in severe harm or death during the study period.

**FIGURE 2:**
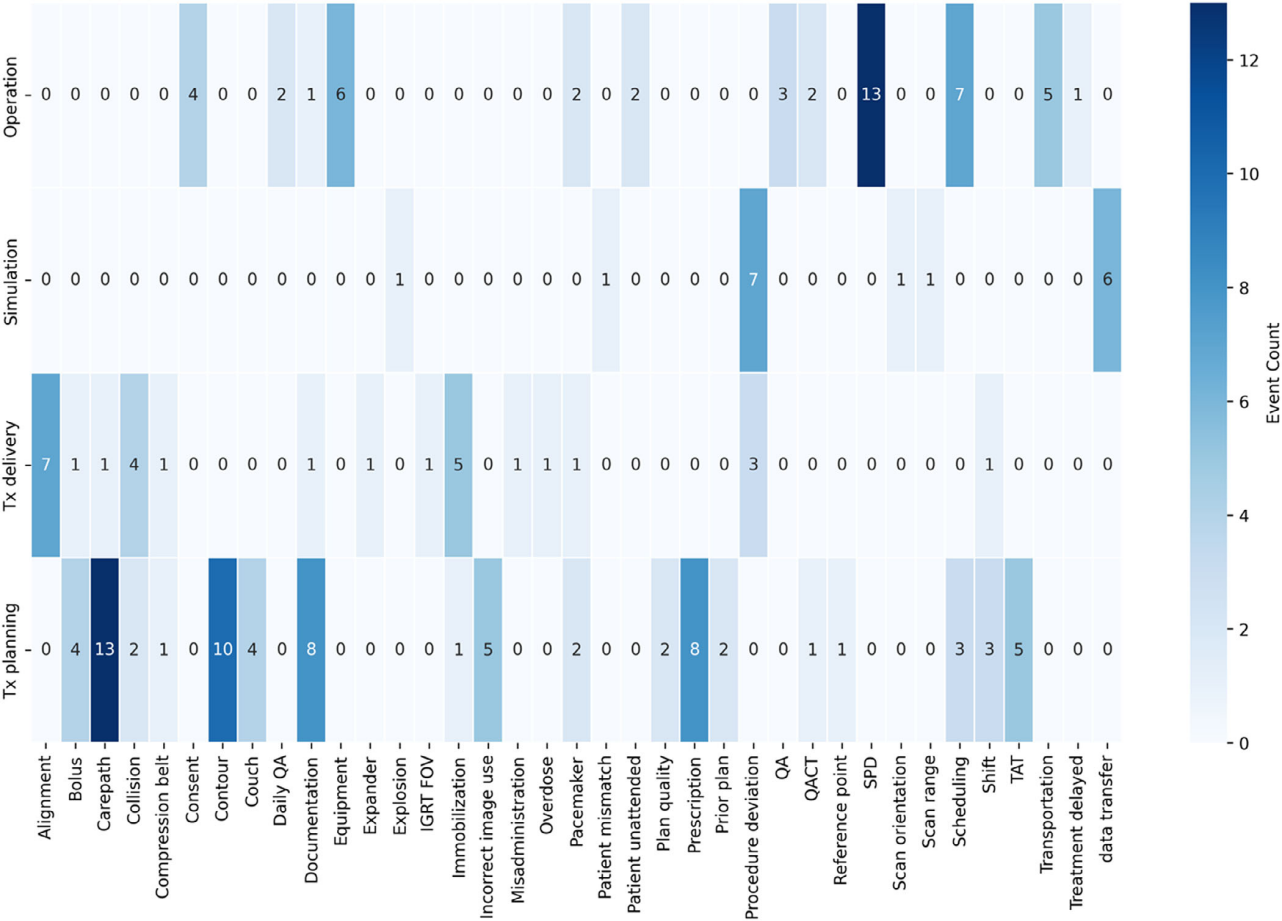
Keywords summary. Incident types were categorized according to Table 1. The keyword “SPD” refers to the Sterilization Processing Department; “TAT” refers to Turn Around Time. The “Others” category was excluded as irrelevant to process improvement.

We identified 136 failure modes and 38 unique keywords. Figure 2 displays a collection of keywords compared against the count of incidents. In the operation category, “Sterilization Processing Department (SPD)” and “Scheduling” were the most frequently reported issues with 13 and 7 incidents respectively. In the treatment planning category, “Carepath” recorded 13 repeated incidents, tying as the most frequent keyword. It was followed by “Contour” with 10 incidents and “Documentation” and “Prescription”, each with 8 incidents. Additionally, “Procedure deviation” in simulation and “Alignment” in treatment delivery were the most common keywords in their respective categories, each with 7 incidents.

### 3.2 Multi‐site correlation

**FIGURE 3:**
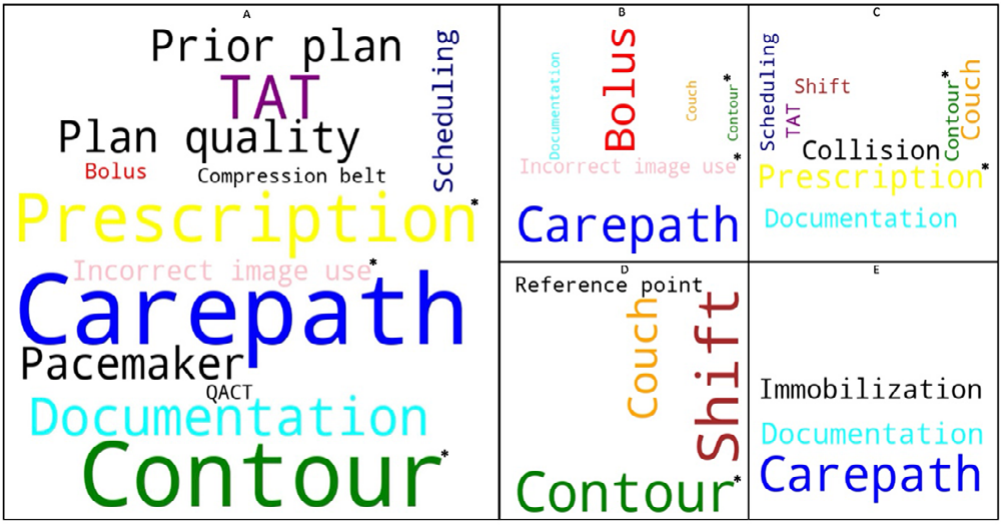
Keywords correlation. Keywords were summarized from five clinical sites (A to E) under the category of treatment planning. “TAT” stands for Turn Around Time. Font size reflects site‐specific frequency, with larger font indicating higher frequency. Keywords with severity greater than 7 are marked with an asterisk (*).

**FIGURE 4:**
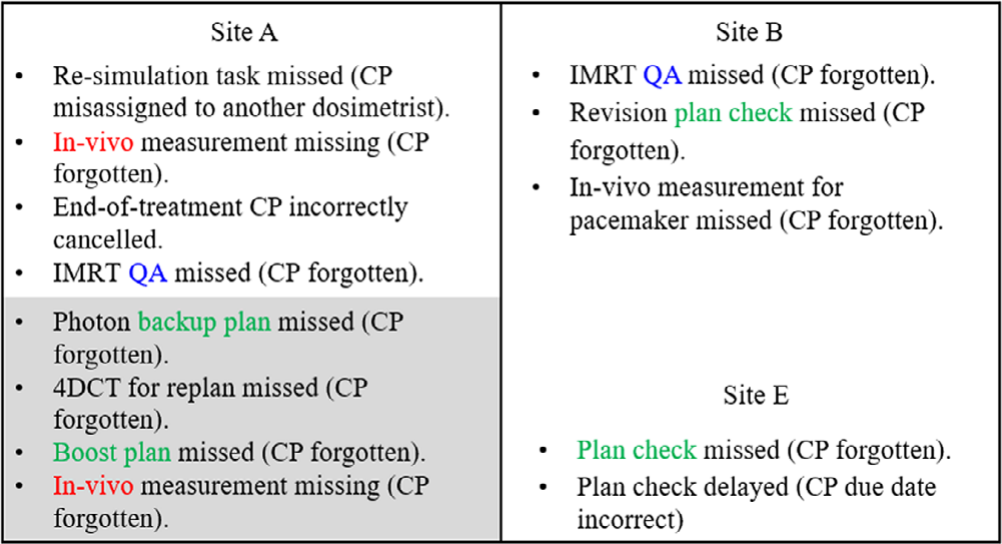
Failure modes for keyword “Carepath (CP)”. Shaded areas indicate failure modes that occurred in proton therapy, and non‐shaded areas indicate those relevant to other modalities (non‐proton therapy).

Treatment planning, which recorded the highest number of incidents was selected for taxonomy analysis, as shown in Figure 3, displaying keyword correlations across multiple sites. The terms “Contour” and “Documentation” were prevalent across four sites, followed by “Carepath” and “Couch”, which occurred in three sites. Notably, “Carepath” appeared most frequently at sites A, B, and E leading to its selection for detailed failure mode comparison as shown in Figure 4.

Figure 4 details the failure modes associated with the keyword “Carepath” with areas relevant to proton therapy shaded. The results showed that ten failure modes were caused by oversights in carepath insertion: four from treatment plans, two from IMRT QA, and two from in vivo dosimetry, occurring in both proton and non‐proton modalities.

### 3.3 Proactive risk assessment

**FIGURE 5:**
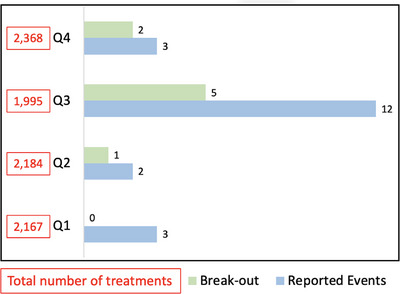
Incident tracking at Site B: Quarters 1 to 4 (Q1 to Q4) are shown. Quarterly data correspond to the study period: Q1 (July to September 2023), Q2 (October to December 2023), Q3 (January to March 2024), and Q4 (April to June 2024). “Break‐out” refers to ad hoc, in‐depth review sessions. Incidents categorized as “Others” are excluded as irrelevant to process improvement.

For Site B, Figure 5 displays the number of treatments for each quarter of the study, along with the corresponding number of reported incidents, excluding events categorized as “Others”, which were not directly related to process improvement. Q3 and the fourth quarter of the study Q4 (April to June 2024) recorded the minimum and maximum numbers of treatments with 1,995 and 2,368 respectively. Conversely, the second quarter of the study Q2 (October to December 2023) and Q3 reported the fewest and most incidents, with 2 and 12 respectively. A total of 20 events were identified. In addition to reviewing all incidents, the committee selected 8 events for further in‐depth analysis due to their complexity.

**FIGURE 6:**
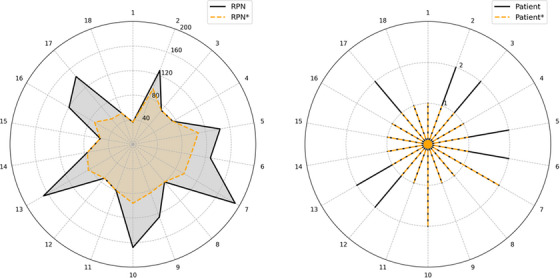
Variations of risk priority number (RPN) and patients. “RPN” and “Patient” represent initial values, and “RPN*” and “Patient*” represent post‐intervention values.

From these reported events, a total of 18 distinct failure modes were derived. Figure 6 quantifies the effectiveness of interventions across the identified failure modes. The left and right graphs illustrate variations in the RPN and the estimated number of affected patients, respectively. Initial values are indicated by solid black lines labeled “RPN” and “Patient”, while post‐intervention values are labeled with dashed orange lines labeled “RPN*” and “Patient*”. The average RPN decreased from 106 to 77, and the total estimated number of affected patients decreased from 27 to 20.

Table 3 shows the outcomes from applying the proactive model to five critical failure modes with a severity exceeding 8. The interventions reduced the average Occurrence (O) from 3.4 to 2.6 (O*) and Detectability (D) from 6.6 to 4.4 (D*). Consequently, the average RPN decreased from 155 to 90 (RPN*), and the predicted number of patients affected (*N*) reduced from 2 to 1 (N*).

**TABLE 3:** Application of proactive model to mitigate critical failure modes.

	Failure mode	O	D	RPN	N	Interventions	O*	D*	RPN*	N*
1	CT scan mislabeled (*S* = 9)	2	8	144	2	‐ Staff increase ‐ Timeout policy re‐enforce	2	6	108	1
2	Patient scan misorientation (*S* = 8)	2	8	128	2		2	6	96	1
3	Wrong image set for treatment plan (*S* = 8)	7	3	168	2	‐ Staff increase ‐ Add checklist items	4	3	96	1
4	Prone belly board misposition (*S* = 8)	4	6	192	2	‐ In‐service training ‐ Add a policy for extended FOV in IGRT	3	4	96	1
5	Unapplied shifts during treatment delivery (*S* = 9)	2	8	144	2	‐ Staff increase ‐ Add machine interlocks	2	3	54	1

O, D, RPN, and N represent initial values; O*, D*, RPN*, and N* represent post‐intervention values.

## 4 DISCUSSION

From 2023 to 2024, our internal ILS recorded a significant increase in reported incidents, with most concerns attributed to treatment planning and operational challenges. Strong overlaps in underlying failure modes were evident across sites. Additionally, the safety culture at one representative site was notably improved, as evidenced by the variations in incident numbers and the subsequent reductions in the risk priority numbers and the number of affected patients, as projected by the proactive model.

The statistical and root cause analyses in our study synergized to establish a system‐wide approach for risk management with optimized resources.^22^ Our initial analyses pinpointed primary concerns through high‐frequency keywords within event categories, as depicted in Figures 1 and 2. Subsequent analyses emphasized keyword correlations, highlighted in Figure 3. Similar taxonomy‐based analysis has proven effective in previous research.^23^ Our workflow minimized redundant efforts on recurring events across multiple sites and facilitated the sharing of site‐specific learnings. The adoption of word clouds enabled better visualization of root causes. Our incident learning was also generalized across modalities, as shown in Figure 4, which collectively enabled the generation of preventative actions, leading to enhanced system‐wide preparedness and management efficiency.

Measurable endpoints are essential for quantifying the impact on safety improvements.^10^ Clark et al.^2^ and Hartvigson et al.^3^ evaluated the effectiveness of various statistical approaches’ endpoints, underscoring the critical role of a positive safety culture where employees feel empowered to report incidents without fear of retaliation. In our study at Site B, we observed a notable trend in Figure 5: incident numbers increased significantly from Q1 to Q3, peaking in Q3 despite the lowest treatment numbers, and then markedly decreased from Q3 to Q4, despite the highest treatment numbers. While more reported incidents may not directly reflect improvements, as discussed by Terezakis et al.,^10^ the observed variation from Q1 to Q4 is a measurable endpoint that suggests a positive cultural shift. The rise in reporting through Q3 reflects growing familiarity and awareness. The Q4 decline was likely driven by breakout reviews and other interventions, including policy changes, in‐service training, and expanded staffing (Table 3). In particular, staff coverage was enhanced with the permanent addition of one radiation therapist (10 years of experience), one therapist assistant (2 years of experience), and one dosimetrist (20 years of experience). Collectively, these measures reinforced safer practices and contributed to the observed reduction.

Besides tracking incidents, the effectiveness of interventions can be benchmarked by the proactive model. Based on Table 2 in TG‐100^12^, Kornek et al. predicted variations in risk priority numbers and the number of potentially affected patients from existing incidents.^19^ We adapted this concept to predict the site‐specific values, as illustrated in Figure 6 and Table 3. As discussed previously, site culture can vary significantly, therefore, absolute values of risk priority numbers or incident numbers may not provide fair endpoints for cross‐site comparisons. Instead, the number of affected patients serves as a more meaningful metric, which can be linked to the estimated costs of interventions,^20^ making the outcomes easier for multidisciplinary leadership team members to interpret and act upon.

Based on the findings of this study, some future directions can be considered. First, it is recommended to record site‐specific data and conduct the hybrid analyses to unbiasedly benchmark safety culture baselines, followed by sharing the results routinely at staff meetings to enhance safety awareness, promote cross‐site preparedness, and increase staff motivation and engagement in safety culture improvements. Second, lessons from existing incident learning can be proactively integrated when implementing new modalities. This approach will particularly benefit the modalities with complex workflows, such as adaptive radiation therapy, which demand significant resources in risk management. Lastly, it is advisable to extend data tracking over extended periods, enabling ongoing refinement and validation of the proactive models and promoting the sharing of institution‐specific data within the broader radiation oncology community.

## 5 CONCLUSIONS

We developed a comprehensive, system‐wide approach that enhanced efficiency in incident learning across multi‐site clinics. This hybrid method, integrating statistics and root cause analysis, effectively benchmarked site‐specific shifts in safety culture and facilitated cross‐site, cross‐modality learning with measurable endpoints. The innovative taxonomy analyses enhanced root cause visualization, pinpointed multi‐site correlation, and conserved resources. Additionally, the proactive approach promoted preventive measures, thereby increasing preparedness throughout the network and collectively leading to improved patient safety outcomes.

## DECLARATION OF GENERATIVE AI AND AI‐ASSISTED TECHNOLOGIES IN THE WRITING PROCESS

During the preparation of this work the author(s) used ChatGPT 5.0 for grammar checking. After using this tool/service, the author(s) reviewed and edited the content as needed and take(s) full responsibility for the content of the publication.

## AUTHOR CONTRIBUTION

**X.L**.: Designed the study; collected and analyzed data; and drafted the manuscript. **F.S**.: Contributed to study design: data review; and manuscript revisions. **Q.L**.: assisted with manuscript review and edits. **J.F., A.C., and A.M**. reviewed the collected data. **R.R**.: supervised the study design and manuscript writing.

## CONFLICT OF INTEREST STATEMENT

An evaluation license for the myQA PROactive software was provided by IBA Dosimetry GmbH. Beyond this, the authors declare no conflicts of interest.

## Data Availability

Research data are stored in an institutional repository and will be shared upon request to the corresponding author.
